# From Nano-Gels to Marine Snow: A Synthesis of Gel Formation Processes and Modeling Efforts Involved with Particle Flux in the Ocean

**DOI:** 10.3390/gels7030114

**Published:** 2021-08-09

**Authors:** Antonietta Quigg, Peter H. Santschi, Adrian Burd, Wei-Chun Chin, Manoj Kamalanathan, Chen Xu, Kai Ziervogel

**Affiliations:** 1 Department of Marine Biology, Texas A&M University at Galveston, Galveston, TX 77553, USA; manojka@tamug.edu; 2 Department of Marine and Coastal Environmental Science, Texas A&M University at Galveston, Galveston, TX 77553, USA; santschi@tamug.edu (P.H.S.); xuc@tamug.edu (C.X.); 3 Department of Marine Science, University of Georgia, Athens, GA 30602, USA; adrianb@uga.edu; 4 Department of Bioengineering, University of California, Merced, CA 95343, USA; wchin2@ucmerced.edu; 5 Institute for the Study of Earth, Oceans and Space, University of New Hampshire, Durham, NH 03824, USA; Kai.Ziervogel@unh.edu

**Keywords:** DOM, marine microgels, marine snow, polymer networks theory, biopolymer self-assembly, primary production, phytoplankton secretion, microbial loop, mathematical modeling

## Abstract

Marine gels (nano-, micro-, macro-) and marine snow play important roles in regulating global and basin-scale ocean biogeochemical cycling. Exopolymeric substances (EPS) including transparent exopolymer particles (TEP) that form from nano-gel precursors are abundant materials in the ocean, accounting for an estimated 700 Gt of carbon in seawater. This supports local microbial communities that play a critical role in the cycling of carbon and other macro- and micro-elements in the ocean. Recent studies have furthered our understanding of the formation and properties of these materials, but the relationship between the microbial polymers released into the ocean and marine snow remains unclear. Recent studies suggest developing a (relatively) simple model that is tractable and related to the available data will enable us to step forward into new research by following marine snow formation under different conditions. In this review, we synthesize the chemical and physical processes. We emphasize where these connections may lead to a predictive, mechanistic understanding of the role of gels in marine snow formation and the biogeochemical functioning of the ocean.

## 1. Introduction

**Figure 1:**
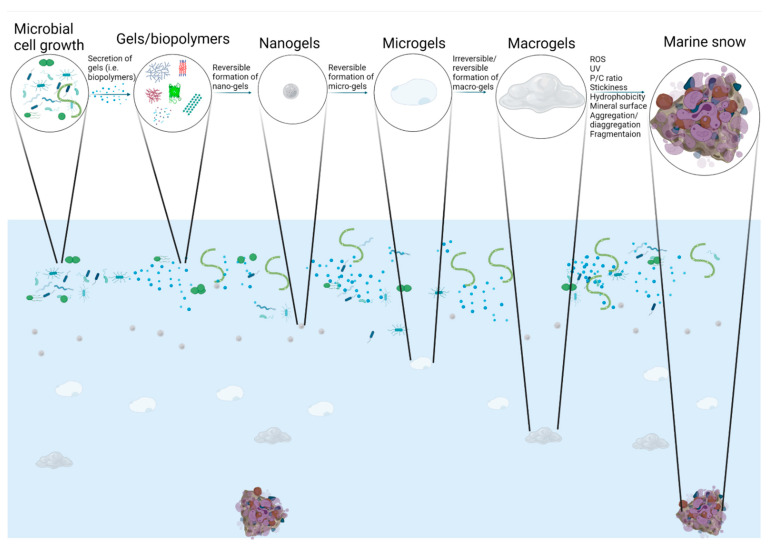
A conceptual model of marine snow formation that requires the following steps: (1) microbial cell growth; (2) secretion of gels (i.e., biopolymers) aka polymeric substances from microbial cells; (3) formation of exopolymeric substances (EPS) which have a variety of forms including TEP and CSP; (4) reversible formation of nano-gels; (5) reversible formation of micro-gels; (5) reversible or irreversible formation of macro-gels; (6) apparent stickiness of particle population dependent on their protein content, i.e., their protein-to-carbohydrate (P/C) ratio; (7) irreversible chemical crosslinking of proteins in gels to form marine snow through hydrophobic or reactive oxygen species (ROS) mediated chemical crosslinking; (8) UV oxidation; (9) interactions of mineral surfaces with gels or marine snow; and (10) aggregation-disaggregation/fragmentation rates. Nanogels (100–150 nm) < microgels (~5 µm) < macrogels (100 µm) < marine snow (>500 µm to 10s of cm) occur on a size continuum.

Global biogeochemical cycling of carbon, nitrogen, and other macro- and micro-elements occurs throughout the water column of the oceans. A fraction of the photosynthetically produced carbon in the sunlit photic zone is modified by biotic processes viz the microbial loop and the biological pump [1,2,3,4,5,6,7]. Up to 50% of the organic carbon produced by phytoplankton is thought to be taken up by bacteria, which are subsequently grazed by nanoplanktonic heterotrophic flagellates that drive the flux of material and energy into the food chain [3,6,8]. Bacteria, which solubilize particles and acquire dissolved organic carbon (DOC) and inorganic nutrients, are then grazed upon by protozoa, and are subsequently preyed on by mucus net-makers and small zooplankton, the latter of which function as conduits to higher trophic levels. In this way, the passively settling particles below the photic zone, known as marine snow (Figure 1), are regarded as a primary source of substrate that supports heterotrophic food webs [9,10]. The vertical flux of carbon and nutrients relies on sinking particles [11]. The flux of particulate organic carbon (POC) through sinking marine snow from surface waters declines exponentially due to consumption, with only 1% of the sinking organic material reaching the seafloor [12].

There is a complicated relationship between DOC and POC, with studies showing a dynamic equilibrium between free and assembled DOC occurring over the whole water column that produces micron-scale gel patchiness that may help to explain carbon turnover, particularly in the dark ocean [13,14]. In addition, Arrieta et al. [15] found that DOC is as readily consumed by bacteria in the surface as in the deep ocean; with rates constrained only by the availability of these materials. A generic relationship between DOC and organic biopolymers forming exopolymeric substances (EPS) [16,17] or transparent exopolymeric particles (TEP) [5,18,19,20] and larger marine snow composites [21,22,23] has been suggested [24] but not yet objectively verified. The goal of this review is to synthesize historical and recent literature to examine the relationship (if one exists) between biopolymers released by microbes and marine snow (Figure 1). This is one of the major gaps in our understanding of the mechanisms that lead to marine snow formation and our ability to accurately model particle processes and fluxes in the ocean. Recent studies suggest developing a (relatively) simple model that is tractable and related to the available data; this will enable us to step forward into new research by following marine snow formation under different conditions. This is critical given the variety of anthropogenic factors that are modifying biogeochemical cycles in the marine environment, specifically those whose fate and transport is intrinsically linked to marine snow formation. This includes but is not limited to engineered nanoparticles (e.g., [25,26]), oil spills and dispersants (e.g., [27,28,29,30]), and nano- and micro-plastics (e.g., [31,32,33,34]). In the recent literature, we often *now* see reference to marine oil snow (MOS; [29,35]) and marine plastic snow (MPS; [36,37]) reflecting the increased awareness and studies in this important arena. This review is not intended to be comprehensive but rather a synthesis of studies across a variety of fields. The reader is therefore referred to the many reviews on gels and their role in the ocean’s carbon cycle if that is their specific interest (e.g., [4,5,7,13,14,21,23,30,38,39,40,41,42,43]).

## 2. Colloidal Nanogels (or Macromolecules) and Microgels

Riley’s [44] early observations of particle formation in seawater pointed to the idea of a reversible exchange between dissolved organic matter (DOM) and particulate organic matter (POM). It is now known that the oceans hold approximately 700 Gt of reduced carbon in a variety of forms (Table 1), with approximately 660 Gt C in the form of DOC [45]. A substantial amount of this material is in the form of microscopic gels that are rich in nutrients and readily available to bacterial colonization [4,14,46,47,48,49]. DOM itself remains in reversible assembly/dispersion equilibrium with free biopolymers, forming porous self-assembled microgels. These materials originate in the organic material produced by phytoplankton and bacteria that form 3D polymer-hydrogel networks. Operationally DOM is defined as material that passes through a 0.7 or 0.4 µm pore size filter, and thus includes colloidal particles and macromolecules in the filter-passing fraction. Given the continuum of sizes, ultrafilter-passing molecules (aka the truly dissolved fraction if a 1 kDa membrane is used) are retained by the membrane (if the concentration factor is low) and thus taken as the colloidal fraction. This low molecular weight DOM will have enhanced permeation behavior with increasing concentration factor [50].

Gel formation and stability depends in part on the physical and chemical properties of their constituent polymers. DOM contains polysaccharides (polyanionic) and proteins (polyelectrolytes) that undergo random motions bringing them into contact with each other, thus resulting in the formation of tangled networks called nanogels, 100–150 µm in size [16,17,45]. Nanogels can be stabilized by Ca^2+^ ions, hydrophobic interactions, or crosslinking by chemical bonds. The rates of polymer collisions, and hence of gel formation depends on the concentration of the polymers and their length, such that gel formation rate depends approximately on the square of the polymer length [51,52,53,54]. Gels also disperse through the process of reptation [55]. This occurs when polymers within the gel axially diffuse out of the gel network, thereby becoming free polymers again. This process occurs on timescales that vary with the length of the polymer so that gels formed by long polymers are more stable [56]. Consequently, factors such as UV radiation that can affect polymer length also impact gel formation and stability and the dynamic equilibrium between polymers and gels. The nonlinear behavior of gel formation is described and predicted by polymer gel physics [41,46,49,56,57,58].

Once nanogels have formed in the oceans, they can interact with each other to form larger, supramolecular networks or microgels (Figure 1) which are ~4–5 µm in size [4,46,58,59] or 3D polymer hydrogel networks [16,60,61] known as physical gels. The latter are stabilized by hydrophobic or ionic bonds as gels are mostly water and so they can interpenetrate [14,49]. This process also leads to a dynamic equilibrium: removal of these “products”, i.e., equilibrium goes right for the larger gels (Figure 1), resulting in the formation of new microgels from nanogels, which in turn will lead to the formation of new nanogels as long as there is sufficient concentration of polymers to support their formation. This process also leads to a dynamic equilibrium such that removal of these larger gels will result in the formation of more microgels from nanogels, which in turn will lead to the formation of more nanogels so long as there is sufficient concentration of polymers to support their formation.

Microgel assembly follows a characteristic second-order kinetics with a thermodynamic yield at equilibrium of approximately 10% of the oceans DOC stock, that is, a 10^4^ increase of local concentrations of organic material or an estimated 70 Gt of carbon in seawater [14]. Temperature, pressure, and pH, which can vary widely in the ocean, affect DOM self-assembly [14], but salinity does not. Orellana et al. [60] found the polymers in the Artic dissolved organic pool assemble faster and with higher microgel yields than at other latitudes. An important property of gels is that they can undergo phase transitions stimulated by changes in environmental parameters [61]. The presence of molecules such as dimethylsulfoniopropionate and dimethyl-sulfide were found to be critical in the Artic [60]. Changes in these parameters lead to volume phase-transitions (e.g., swelling or dehydration and condensation of the polymer network), collapsing the gel into a denser polymer network. This process can lead to small molecules or even proteins being trapped within the collapsed gel and potentially being transported to depth [62]. The reversible phase transitions shown by these marine gels, as a function of pH, dimethyl-sulfide and dimethylsulfoniopropionate concentrations, can reduce the gel size to <1 μm in diameter [45,60].

Short polymers form only nanogels that remain in continuous assembly/dispersion equilibrium. Dynamic light scattering and other techniques for measuring microgels (up to ~5 µm) have to utilize prefiltered (0.5 µm size) seawater as larger particles interfere. Nevertheless, direct evidence of the critical importance of polymer size on assembly is illustrated by the observation that UV cracking of DOM polymers results in shorter chains, longer assembly times, and smaller-size gels [59]. However, recent studies suggest that if natural sunlight is used, aggregation of EPS by reactive oxygen species (ROS) mediated chemical crosslinking of the protein fraction and microgel formation occurs in parallel to fragmentation and degradation [63,64,65,66].

The mix of biopolymers in these gels collectively referred to as EPS includes predominantly polysaccharides (neutral carbohydrates, amino sugars) and proteins [67,68], with nucleic acids and lipids present but at significantly lower concentrations. The distinctive sugar and amino acid compositions of the colloidal fraction are relatively uniform throughout the ocean such that these chemical signatures are used to test for selective assembly of biomacromolecules into gels [57]. These biopolymers are either released by phytoplankton primary production, bacterial activity, or are the end products of the degraded detritus of marine biota [16,39,59]. Acidic polysaccharides such as uronic acids contain carboxyl groups that provide binding sites for divalent (e.g., Ca^2+^, Mg^2+^) or trivalent (e.g., Fe^3+^) ions providing bidentate inner-sphere coordination sites that can cause supra-macromolecular aggregation and Ca^2+^ bridging for structural stability [4]. Proteins, as another major EPS component, are amphiphilic and mediate the stability and aggregation of the 3-D networks of biopolymers, through both hydrophobic and electrostatic interactions [68,69,70], as well as light-induced cross-linking [63,64,65,66]. EPS are often subcategorized into truly dissolved (<1 nm) or colloidal (1–1000 nm) fractions [8,43,58,71,72,73,74,75].

EPS are thought to drive marine particle formation (Figure 1), including marine macrogels and marine snow [9,20,30,74]. EPS may be hydrophobic or hydrophilic [58,75]. Very small amounts of amphiphilic EPS can greatly accelerate micro-gel formation [58]; these in turn have been shown to be biomes for accelerated microbial activity [4,41,76,77,78,79]. Stoderegger and Herndl [80] first introduced the idea that hydrophobic interactions might play an important role in coagulation of marine particles, suggesting that EPS hydrophobic properties might be responsible for POC and bacterial aggregation. Studies monitoring DOM assembly showed that EPS from the marine bacteria *Sagittula stellata* induce microgel formation [58,75] following typical hydrophobic polymer-bonding kinetics. The assembly of DOM polymers and hydrophobic EPS follows a first-order kinetics and requires a much lower concentration that are different from DOM polymers or amphiphilic EPS alone. In addition, *Sagittula* EPS-induced DOM assembly and microgel formation exhibited typical temperature-enhanced cooperativity found in hydrophobic interaction-driven processes with typical high cooperativity [75]. Enhancement of hydrophobic interactions with temperature results from temperature-induced conformational changes of amphiphilic polymers that produce increased hydrophobic contact area [69,81] and a higher probability of inter-chain bonding.

An important additional characteristic of EPS-induced DOM assembly is the critical assembly concentration that remains proportional over a broad range of surfactant concentrations and is independent of both polymer charge and the presence of counter ions [81]. In fact, DOM assembly induced by more hydrophobic EPS (higher protein content) can remain virtually unchanged in Ca^2^^+^-free seawater [58]. The fundamental mechanisms of hydrophobic bonding within EPS, however, remain obscure [30]. What is clear is that the hydrophobic effect is caused by the interaction (aggregation or clustering) of hydrophobic moieties or molecules (exposed after unfolding) when they are surrounded by hydrophilic environment.

Release of EPS may allow bacteria (and phytoplankton) to wrap themselves in a DOM network of virtually locked-up nutrients. DOM becomes a target easily cleaved by bacterial exoenzymes to yield low-molecular-weight oligomers that can be readily imported and metabolized by the bacteria [79,82,83]. This inference agrees with observations that bacteria are found on hot spots [1,8,78,84,85], lodged in and around the EPS-induced DOM networks or colonizing DOM self-assembled networks [48]. EPS release may therefore allow bacteria to concentrate substrate that is otherwise inaccessible at the characteristic low DOM concentration found in seawater (see also [15]). Recent reports that EPS from *Synechococcus*, *Emiliania huxleyi*, and *Skeletonema costatum* can self-assemble in Ca^2+^-free artificial seawater indicate that phytoplankton EPS might also be responsible for the production of the vast majority of microgels [75]. Considering the rich and ubiquitous presence of phytoplankton in the ocean and high-yield secretory activity [36,86,87,88], these results suggest that microalgae might not only be a major source of reduced organic carbon but also may release amphiphiles that can induce DOM assembly.

## 3. TEP and CSP

Marine gels are part of a colloidal continuum that, at larger sizes, may operationally be defined as TEP or Commasie Stained Particles (CSP) because of their gel-like nature. The work by Alice Alldredge and colleagues first described the link between polysaccharide-containing macrogels (TEP; Figure 1) and the flocculation of diatoms resulting in the formation of marine snow [21,89]. The later studies showed that TEP form discrete sheets, films, or strings ranging from three to 100 µm. The promotion of coagulation of TEP provides the matrix for marine snow [4,39,40,89], particularly when it interacts with calcium or silica based biominerals [90,91]. Today we know that TEP are found throughout the water column from the sunlit surface layers to the dark ocean [92].

Busch et al. [93] and Engel et al. [94] showed detailed distributions of polysaccharides and proteins in marine gel particles that were stained as TEP and CSP, respectively. Busch et al. [93] reported that at all stations, their results showed strong positive correlations over depth between gel particle number and total gel particle area for both gel particle types (TEP and CSP), with gel particle diameters of several hundred µm, and bacterial colonization of 1–2 × 10^5^ cells/mm^2^. Average proportions of bacteria attached to gel particles ranged from 1–4%, with most of the bacteria free-living.

Engel et al. [94] state in their paper, “extracting three-dimensional, fractal gel particles onto membrane filters, staining, and subsequent measurement of their two dimensional size introduces inevitable inaccuracies. Likewise, estimating their main chemical components by compound-specific stains that may target one but not all components is prone to error.” Regardless of the potential inaccuracies or biases, these gel particles accounted for about 0.1 to 10% of DOC, depending on the depth and ocean. Thus, it seems to be clear that these large gel particles are important, especially in the surface ocean.

It should be noted that TEP and CSP are measured using chemical stains (Alcian blue or Commasie blue respectively at low pH) that potentially alter the physical and/or chemical structures of marine organic matter. These induced alterations discourage further investigation of gel physicochemical characteristics for microgels as described in Chin et al. [46]. Measurements of TEP, EPS, and microgels were recently evaluated in Xu et al., [67]. While there was reasonable agreement or significant relationships between each of the three operational methods, i.e., TEP, EPS, and gels; they are not completely equivalent. Although these terms are often used interchangeably, they are mostly operational definitions given their respective quantification methods [30]. Measurements from one method cannot be directly applied to or substitute for the others. This clarification is critical as it has not been shown that these rigidly fixed and stained particles have the emergent physicochemical properties of gels as defined by Chin et al. [46] using polymer physics approaches. Nor has it been clarified if any staining method might even induce aggregation by crosslinking some macromolecules. An awareness of these differences is fundamentally critical because it affects future modeling efforts (see Section 5 below) if we expect them to be mechanistic and predictive.

## 4. Marine Snow

How marine snow-sized (particles >500 µm in length) macrogels reach their large final equilibrium size has not been rigorously established (Figure 1). It is likely that they undergo multiple annealing steps whereby interpenetration by elongated undegraded polymers allows these gels to reach a larger, more stable equilibrium size [20,30]. Conversely, assembly of shorter chains from the DOM fraction yields a correspondingly large pool of gel that can self-assemble throughout the whole water column [49].

The gel component of detrital marine snow particles can determine the properties of these particles. For example, Alldredge and Gottschalk [95] determined that marine snow sinking rates were non-Stokesian. Sinking rates were not related to excess density, but to diameter as a power function, with an exponent of about 0.26. In Alldredge et al. [96], they established that marine snow particle aggregate size was related to TEP content. This is likely a result of the positive buoyancy of TEP reducing the excess density of the particle, with larger aggregates containing more TEP and thereby reducing the sinking speed of the aggregates [5].

Changes in surface tension due to emulsifier or EPS addition to seawater also affect or control aggregation processes. Schwehr et al. [81] using model EPS constituents, such as protein (BSA, bovine serum albumin) and uronic acids (glucuronic acid with carboxyl moieties; carrageenan with sulfate groups), showed that increasing the protein to carbohydrate (P/C) ratio of EPS lowers the surface tension, which in-turn resulted in aggregation through Ca^2+^ bridging. This implies that gel growth and marine snow formation occur through a combination of Ca^2+^ bridging of acid polysaccharides, and ROS-mediated chemical crosslinking of proteins, in parallel to enzymatic cleavage, oxidation, and degradation pathways [66]. Schwehr et al. [81] also showed that surface tension overall was a function of the P/C ratio of the EPS in the water. The implication is that the surface tension reduction is related to the gel formation mechanisms.

The ratio of EPS has been determined to be an important parameter in determining the “stickiness” or attachment propensity or aggregation potential [68,97], thereby regulating the overall DOM-particulate organic matter (POM) continuum [4,98]. Among the aggregation mechanisms that need to be considered, there is the newly discovered coupled process of ROS-mediated chemical crosslinking of proteins under sunlight [63,64,65,66]. These authors demonstrated that sunlight can directly induce aggregation of different kinds of EPS, with greater effect on EPS containing more protein, [65], as the aggregation of EPS is actually caused by aggregation of proteins, in parallel to some cleavage of high molecular weight compounds into smaller, less stable fragments. The findings revealed that the UVB light is of higher energy that can cleave DOM polymers [46,59] and TEP particles [69], and thus reduces their spontaneous assembly. In contrast, the UVA and visible light have lower energy to cleave polymers, but are capable to induce chemical crosslinking of individual macromolecules and marine snow aggregate formation. The increase in particle size of EPS from seven different microbial species was found to be positively correlated to the P/C ratio of EPS [88]. On the other hand, no marine snow aggregates were observed for a non-protein containing EPS from a phytoplankton species [36,88]. It was also shown that hydroxyl radical and peroxide played critical roles in this photo-oxidation process, and ionic strength (controlled by salt concentration) and Ca^2+^-bridging assisted the aggregation process that leads to marine snow formation. The formation of higher molecular weight products compared to the native proteins, with simultaneously increased carbonyl content, was demonstrated by gel electrophoresis. The model proteins ultimately became more resistant to proteolysis [66]. The addition of ROS (i.e., H_2_O_2_ and •OH) only accelerated the observed transformations under simulated sunlight. Sun et al. [66] thus demonstrated that photo-oxidation can transform labile proteinaceous materials into refractory matter, providing a novel mechanism for the preservation of high molecular weight dissolved organic nitrogen in the ocean. These observations provide new insights into polymer assembly, marine snow formation, and the fate/transport of organic carbon and nitrogen in the ocean. The relatively elevated P/C ratio of EPS induced by environmental stresses was found to associate with gel formation [36,88]. The effect of environmental stresses is similar on both phytoplankton and bacteria [88]. The stickiness increase of EPS association with environmental stresses might provide additional insights for the recurrent massive mucus aggregates (sea snot) incidents in Adriatic and Mediterranean Sea [99,100].

Even though the bulk P/C ratios that are used as a proxy for the stickiness of biopolymers that are mainly comprised of the two major components (proteins and carbohydrates) [37,88,97], one still needs to take the individual monosaccharides or amino acid composition (i.e., individual species and their relative abundances) into account for more accurate analysis of the relative hydrophobicity/hydrophilicity of the biopolymers. For example, at neutral pH (6.8), acidic carbohydrates (i.e., glucuronic acid) are negatively charged while amino sugars like glucosamine are positively charged, which results in opposite electrostatic interactions. Another example is the microbial degradation of agal-derived EPS resulted in an increase of the deoxy sugars, fucose, and rhamnose and thus also a possible increase in the hydrophobic features of the EPS due to the carbon six methyl group. Amino acids also have a good grouping according to their hydrophobicity/hydrophilicity scaling [101,102].

A synthesis of the literature, microscopic observations of natural colloids, experimental results obtained with model systems, and numerical simulations, led Buffle et al. [98] to conclude that the formation of aggregates such as marine snow in aquatic systems can be understood by mainly considering the roles of three types of colloids: (i) compact inorganic colloids; (ii) large, rigid biopolymers such as polysaccharides; and (iii) either the soil-derived fulvic compounds or their equivalent in pelagic waters, aquagenic refractory organic matter. In most natural aquatic systems, the small (few nanometers) fulvic compounds will stabilize the inorganic colloids whereas the rigid biopolymers (0.1–1 μm) will destabilize them, i.e., lead to floc (marine snow) formation. The concentration of stable or unstable (i.e., aggregated) colloids in a particular aquatic system will depend on the relative proportions of these three components. Santschi et al. [17] showed experimental evidence that the number of marine snow particles >1.5 mm (imaged by a well-calibrated camera system) in the Middle Atlantic Bight, ranged between 5 and 40 aggregates/L. The abundance of marine snow aggregates (but not the suspended particle concentration) were well correlated with the deficiency of a short-lived particle-reactive radionuclide, Th-234, with respect to its production rate by its long-lived radioactive parent, U-238, down to 2500 m water depth, indicating the strong scavenging ability of these marine snow particle aggregates.

## 5. Modeling Efforts

Basin-wide and global biogeochemical models of oceanic carbon export often explicitly include DOM, but do not unequivocally include gels or TEP, as the latter two terms are more varied in the field or laboratory collection and detection methods (see above, 67 and references therein) though some take steps to incorporate simplified representations. One of the first numerical models on aggregate formation in aquatic systems was published by Jackson [103] where he combined kinetic coagulation theory with simple algal growth kinetics to describe the dynamics of an algal bloom. Results of his model show that coagulation, and subsequent sinking of aggregates, dominates the dynamics when algal concentrations are above a certain critical value [103,104]. This critical algal concentration varies inversely with the fluid shear, algal size, and a parameter called the “stickiness”. The stickiness, or coagulation efficiency, represents the probability that two particles will adhere once they have collided, and it is through this parameter that the effects of gels can potentially be incorporated. However, it is important to note that at present there has been little effort into making the stickiness more than a fudge factor, whereas in reality it is likely related to the formation and properties of EPS, gels, and TEP.

Jackson [105] used his previous coagulation model to examine the results of a mesocosm experiment suggesting that aggregating TEP particles with algal particles changes the overall stickiness of the aggregates. However, it was unclear if changes in the TEP-algal particle interactions resulted from the inherent stickiness of TEP or from the increased particle concentration that resulted from explicitly including the TEP particles in the model. Mari and Burd [18] adapted Jackson’s model to explicitly model the coagulation of algal cells with TEP using measured TEP size distributions, but even there, stickiness parameters for the interaction between TEP and algal particles were held fixed and were not based on any underlying physico-chemical processes. Oguz [106] took a different approach by explicitly modeling the formation of TEP from DOM secreted by phytoplankton and bacteria, but prescribing aggregation rates rather than determining them using coagulation theory. More complicated models of marine snow formation have been used that include simplified models of TEP formation and particle aggregation [107,108]. However, even in these models, the stickiness of particles is prescribed and not based on any fundamental quantity such as the P/C ratio which may provide a way of improving aggregation models.

More recently, the PISCES biogeochemical model [109] includes a single, semi-labile DOC pool formed from bacterial, phytoplankton secretion, and zooplankton excretion. The DOC can aggregate with itself to form POC, and it can aggregate with POC. Aggregation in this model assumes that particles obey a steady-state power-law size distribution, which makes the models computationally efficient, but does not fully capture the reversible equilibrium dynamics between DOC, gels, and POC. On the other hand, Maerz et al. [110] modeled particle fluxes in the global ocean using the latest algorithms, called “novel Microstructure, Multiscale, Mechanistic, Marine Aggregates in the Global 5 Ocean (M4AGO) sinking scheme”. In their model, they related adhesion properties, i.e., particle stickiness, to the fractal nature of the aggregates, assuming that the stronger the surface adhesive forces are, the higher the stickiness of particles. Accompanying this, the intrusion of particles and particle clusters into each other would also diminish resulting in a looser structure with smaller fractal dimension.

## 6. Marine Gels and the Ocean’s Carbon Cycle

Massive sedimentation events of marine snow have often been observed at the decline of a phytoplankton bloom when the EPS production increases in response to nutrient stress [39,111,112]. Sinking marine snow ultimately removes CO_2_ from the atmosphere, thus balancing the atmospheric carbon levels on geologic timescales [12,113]. Quantifying vertical fluxes of POM in the ocean is however, complicated by seasonal and inter-annual variations that determine marine snow formation and sedimentation [114,115]. This in turn alters the quality and quantity of POM, and settling rates, which are a function of the size and density of marine snow [42]. Particulate inorganic matter including clay minerals from terrestrial sources as well as carbonate sheathes in the form of calcite foraminifera shells, coccolith plates, and aragonite pteropod shells and shell fragments, may act as ballast for marine snow increasing its density and thus sinking rates through the water column [11,116]. Additional factors affecting the fate of sinking marine snow include microbial decomposition and grazing as well as physical fragmentation during transit [117,118].

Climate change and related acidification and warming of the surface ocean affect microbial metabolic rates including the release of extracellular organic compounds that form particulate EPS [119,120,121,122,123] and, as a consequence, marine snow sedimentation and carbon sequestration at depth in the future ocean [124]. On the other hand, accelerated microbial oxidation rates of EPS in a warming ocean may counteract carbon export fluxes to the deep sea, preserving more organic matter in surface waters [120,123]. EPS dynamics in a warmer and more acidic ocean have been found to also depend on other environmental factors such as nutrient availability for primary and secondary producers [125,126], complicating future predictions of marine snow sedimentation and thus the efficiency of the biological pump under future climate scenarios.

## 7. Conclusions

The formation of marine snow from biopolymers secreted by microbes is a dynamic progression that relies on biological, physical, and chemical processes. Biopolymers can associate in a reversible manner to form nanogels, which in-turn can reversibly interact with other supramolecular networks (e.g., geopolymers like humic substances, terrestrial origin and more degraded compared to the freshly produced EPS) [127,128] and DOM to form micro and macrogels. Such dynamic interaction between biopolymers, gels and debris along with the influence from physical and chemical processes such as UV and ROS interactions could eventually leads to the formation of marine snow. Furthermore, factors such as biopolymer interactions with divalent and trivalent ions, as well as their composition (P/C), concentration, and length have been shown to play a major role in the transformation of gels to marine snow. Although, the above mentioned processes and factors to some extent explain the relationship between gels and marine snow, much remains unclear. Further studies on marine snow formation that integrates laboratory and in situ aspects along with incorporation of stickiness factor such as P/C ratio of EPS in modelling efforts can further our understanding of the relationship between gels and marine snow.

## Figures and Tables

**Table 1:** Major carbon pools in biogeochemical cycles along with their operational definitions.

Acronym	Carbon Pool	Operational Definition
DOC	dissolved organic carbon	Fraction of organic carbon that passes through a filter (either 0.7 µm GF/F or 0.4 µm polycarbonate filter); It contains polymers (e.g., carbohydrates, proteins) that spontaneously self-assemble; these free biopolymers form nanogels.
DOM	dissolved organic matter	Material that passes through a filter (either 0.7 µm GF/F or 0.4 µm polycarbonate filter); includes colloidal particles and macromolecules.
POC	particulate organic carbon	Fraction of carbon retained by the filter.
POM	particulate organic matter	Fraction of organic matter retained by the filter.
EPS	extracellular polymeric substances or exopolymeric substances	Protein and polysaccharide rich materials with smaller amounts of nucleic acids and lipids.
TEP	transparent exopolymeric particles	Alcian-blue stainable transparent particles that are formed from acid polysaccharides.
CSP	Commassie stained particles	Protein-rich Commassie stainable particles.
	marine snow	Composite particles (algae, bacteria, feces) in a matrix of EPS which is visible to the naked eye.
